# The Use of Oral Analgesics and Pain Self-Efficacy Are Independent Predictors of the Quality of Life of Individuals with Rheumatoid Arthritis

**DOI:** 10.1155/2020/7409396

**Published:** 2020-07-23

**Authors:** Akira Hashimoto, Motoki Sonohata, Masaaki Mawatari

**Affiliations:** Department of Orthopaedic Surgery, Faculty of Medicine, Saga University, Nabeshima 5-1-1, Saga 849-8501, Japan

## Abstract

**Objectives:**

This study investigated the relationship between quality of life (QOL) and several factors, including pain assessments, in patients with rheumatoid arthritis (RA).

**Methods:**

This cross-sectional, single-center study enrolled 85 patients with RA. The variables investigated included demographic characteristics, the 28-joint disease activity score with C-reactive protein (DAS28-CRP), painDETECT questionnaire (PDQ), pain self-efficacy questionnaire (PSEQ), and pain catastrophizing scale (PCS). QOL was measured using the Japanese validated version of the European Quality of Life questionnaire with five dimensions and five levels (EQ-5D-5L).

**Results:**

The use of oral steroids and oral analgesics was significantly associated with low EQ-5D-5L scores (*P* < 0.05). EQ-5D-5L score had a significant positive association with PSEQ (*r* = 0.414) and significant negative association with age, disease duration, DAS28-CRP, PDQ, and PCS (*r* = −0.217, −0.343, −0.217, −0.277, and −0.384, respectively). Multiple regression analysis showed that the use of oral analgesics and PSEQ were independent predictors of EQ-5D-5L score (*β* = -0.248, *P* < 0.05 and *β* = 0.233, *P* < 0.05).

**Conclusions:**

The use of oral analgesics by RA patients may influence their QOL, which, in turn, may affect their feelings of self-efficacy. Various pain management strategies, including surgical treatment, may be explored for the treatment of RA. Furthermore, the PSEQ may be a prominent part of the patient's overall assessment.

## 1. Introduction

Rheumatoid arthritis (RA) is a chronic, progressive, systemic inflammatory autoimmune disease that causes joint deformity, pain, and functional disability [1, 2]. RA affects approximately 0.5–1% of the population globally [3]. The combination of methotrexate and biological disease-modifying antirheumatic drugs (bDMARDs) has contributed to an increase in the number of patients who achieve clinical remission [4]. Disease activity in RA is often assessed using the disease activity score in 28 joints (DAS28) [5], which can further be combined with the C-reactive protein level (DAS28-CRP). However, 12.5% of patients with RA continue to report clinically significant pain despite achieving remission according to the DAS28-CRP score for more than 1 year [6]. A previous report examining patients with RA found that even those with low disease activity may have neuropathic pain [7]. Pain is usually associated with poor quality of life (QOL) in patients with RA [8].

Self-efficacy beliefs and pain catastrophizing also affect QOL in patients with RA [9, 10]. Satisfactory QOL is an important component of remission; complete remission of RA is defined as the achievement of clinical, structural, and functional remission [11]. Therefore, the main goal of RA treatment is the improvement of the patient's QOL [12]. However, the overall benefit of intensive treatment strategies in RA remains uncertain [13].

One of the common health-related QOL (HRQOL) measurement tools is the European Quality of Life questionnaire with five dimensions and five different response options (EQ-5D-5L) [14–16]. Several reports have described the use of the EQ-5D-5L for assessing the QOL of patients with RA [17–19]. However, those reports included a limited number of factors [17–19], and none have investigated the relationship between the current study variables, such as pain self-efficacy or pain catastrophizing, and QOL using the EQ-5D-5L.

In this study, we aimed to investigate the variables that influence the QOL of patients with RA, using the EQ-5D-5L.

## 2. Materials and Methods

### 2.1. Patients and Methods

This single-center study utilized a cross-sectional design and was conducted at our institution in October 2016. The study protocol adhered to the ethical guidelines of the 1975 Declaration of Helsinki; the study design was approved by the appropriate ethics institutional review board (No. 2016-02-02), and all study participants provided informed consent.

A total of 87 patients with RA were recruited for this study. Two patients were excluded from the analyses due to incomplete data, and 85 patients were included in the final analyses. Aside from a lack of data, there were no other exclusion criteria. All patients had a confirmed diagnosis of RA according to the 2010 American College of Rheumatology/European League Against Rheumatism [20]. The study variables included age, sex, body mass index (BMI), disease duration, presence and number of RA-related operations, use of MTX, use of bDMARDs, use of oral steroids, use and type of oral analgesics, use of synthetic DMARDs (sDMARDs) in patients with oral analgesics, serum matrix metalloprotease-3 (MMP-3) levels, EQ-5D-5L, DAS28-CRP, painDETECT questionnaire (PDQ), pain self-efficacy questionnaire (PSEQ), and pain catastrophizing scale (PCS).

### 2.2. Measurement and Assessment Tools

#### 2.2.1. EQ-5D-5L

The EQ-5D-5L is a common HRQOL measurement tool. The EQ-5D-5L measures HRQOL using five items: mobility, self-care, usual activities, pain/discomfort, and anxiety/depression [15]. Each item has five different response options: no problems, slight problems, moderate problems, severe problems, and unable to/extreme problems; the combination of responses yields 3125 unique health statuses [15]. A Japanese version of the EQ-5D-5L has been developed [16]. The total scores range from 0 to 1 [16], and a higher score indicates a better QOL.

#### 2.2.2. DAS28-CRP

The DAS28-CRP is an assessment of disease activity in RA. DAS28-CRP scores are calculated by assessing three components: (1) the number of swollen and tender joints, out of 28, as determined by a trained rheumatologist; (2) the patient Visual Analog Scale for general disease activity; and (3) CRP levels [5, 21]. RA activity is categorized according to the DAS28-CRP score as remission (<2.3), low (2.4–2.7), moderate (2.8–4.1), or high (>4.1) [22]. In this study, DAS28-CRP was evaluated as a continuous variable, without dividing it into categories.

#### 2.2.3. PDQ

The PDQ is an assessment for measuring noninflammatory, neuropathic, or sensitization elements of pain [23]. The PDQ contains seven items evaluating pain qualities, one evaluating the course pattern of pain, and one evaluating pain radiation [23]. The questionnaire contains three 0–10 numerical rating scales for current, worst, and average pain severity. An overall score is generated, which summarizes everything but the pain intensity numerical rating scales, and this overall score ranges between −1 and 38. An overall score >18 indicates likely neuropathic pain, 13–18 possible neuropathic pain, and <13 unlikely neuropathic pain [23]. In this study, PDQ was evaluated as a continuous variable.

#### 2.2.4. PSEQ

The PSEQ is an assessment of the patient's confidence in performing activities despite pain [24]. It is a 10-item questionnaire; each item is rated on a 7-point numerical rating scale. A higher score indicates higher self-efficacy or more confidence in managing chronic conditions. The total scores range from 0 to 60. The Cronbach's *α* coefficient for the Japanese version of the PSEQ is 0.94 [25].

#### 2.2.5. PCS

The PCS is an assessment of the degree of catastrophic thinking regarding pain. It comprises 13 items, each of which is rated on a 5-point numerical rating scale. The PCS has three subscales: rumination, magnification, and helplessness [26]. Higher scores indicate greater levels of catastrophizing. The total scores range from 0 to 51. The Cronbach's *α* coefficients for the Japanese version of the PCS are 0.80 for the rumination subscale, 0.65 for the magnification subscale, 0.81 for the helplessness subscale, and 0.89 for the total score [27].

### 2.3. Statistical Analysis

All numerical data were expressed as the mean ± standard deviation. All statistical analyses were performed using SPSS version 23 for Windows (International Business Machines Corporation (IBM), NY, USA). The Kolmogorov–Smirnov test was conducted to evaluate the distribution normality of the continuous variables. Independent *t*-tests were used to compare the EQ-5D-5L scores by sex, history of any RA-related operations, use of MTX, use of bDMARDs, use of oral steroids, and use of oral analgesics. Independent *t*-tests were also used to compare the use of oral analgesics with the individual items of the PSEQ. Pearson correlation coefficients were obtained to assess the correlations between the EQ-5D-5L and age, BMI, disease duration, number of RA-related operations, MMP-3 levels, DAS28-CRP, PDQ, PSEQ, and PCS; between DAS28-CRP, PDQ, PSEQ, and PCS; and between EQ-5D-5L and the score of each of the items of the PSEQ. Tukey's test was used to compare the scores among the 10 items of the PSEQ.

We performed a multivariate analysis to identify the variables that independently predict the EQ-5D-5L scores using multiple linear regression [28, 29]. The variables included in the multivariate analysis were those with a *P* value < 0.05 in the univariate analyses [28, 29]. The level of significance was set at *P* < 0.05.

## 3. Results

The demographic characteristics of the patients are shown in Table 1. The type of oral analgesics used and the percentage of patients who also use sDMARDs in addition to oral analgesics are shown in Table 2. The percentage of patients who used oral analgesics was 29.4%. The overall percentage of patients who use sDMARDs out of all patients who use oral analgesics was 92.0%. The percentage of patients who use sDMARDs out of all patients who use nonsteroidal anti-inflammatory drugs (NSAIDs) was 94.7%.

**Figure 1:**
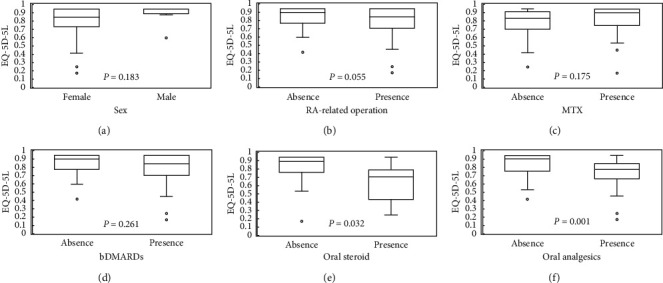
Box-and-whisker plots showing differences in EQ-5D-5L for the dichotomous study variables. (a) Sex, (b) history of any RA-related operations, (c) use of MTX, (d) use of bDMARDs, (e) use of oral steroids, and (f) use of oral analgesics. Data are presented as the mean ± standard deviation. EQ-5D-5L: the European Quality of Life questionnaire, five dimensions, five levels; RA: rheumatoid arthritis; MTX: methotrexate; bDMARDs: biological disease-modifying antirheumatic drugs.

The results of the independent *t*-tests between the EQ-5D-5L and the studied variables are shown in Table 3 and Figure 1. The use of oral steroids and oral analgesics was significantly associated with low EQ-5D-5L scores (both *P* < 0.05).

**Figure 2:**
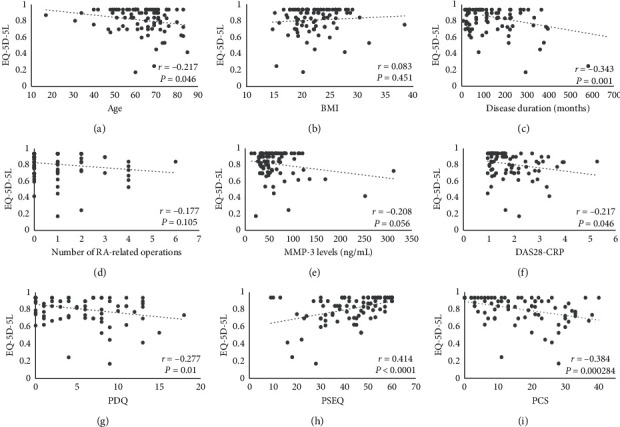
Correlations between the EQ-5D-5L and continuous study variables. (a) Age, (b) BMI, (c) disease duration, (d) number of RA-related operations, (e) MMP-3 levels, (f) DAS28-CRP, (g) PDQ, (h) PSEQ, and (i) PCS. EQ-5D-5L: the European Quality of Life questionnaire, five dimensions, five levels; BMI: body mass index; RA: rheumatoid arthritis; MTX: methotrexate; MMP-3: serum matrix metalloprotease-3; DAS28-CRP: the disease activity score based on the 28-joint assessment-C-reactive protein; PDQ: painDETECT questionnaire; PSEQ: pain self-efficacy questionnaire; PCS: pain catastrophizing scale.

Pearson correlation coefficients between the EQ-5D-5L and studied variables are shown in Figure 2. The correlations were significant for age, disease duration, DAS28-CRP, PDQ, PSEQ, and PCS (*r* = −0.217, −0.343, −0.217, −0.277, 0.414, and −0.384, respectively). Results of the multiple regression analysis between the EQ-5D-5L and study variables are shown in Table 4. Among these, the use of oral analgesics and PSEQ scores were independent predictors (*β* = −0.248, *P* < 0.05 and *β* = 0.233, *P* < 0.05).

**Figure 3:**
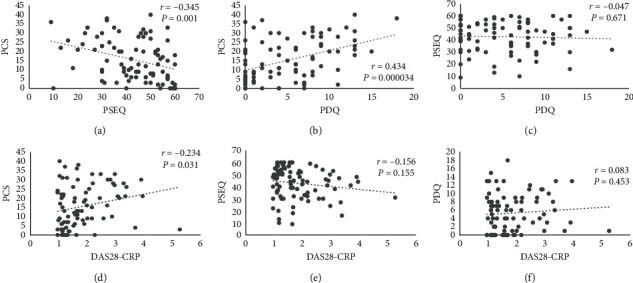
Correlations among the DAS28-CRP, PDQ, PSEQ, and PCS. DAS28-CRP: the disease activity score based on the 28-joint assessment-C-reactive protein; PDQ: painDETECT questionnaire; PSEQ: pain self-efficacy questionnaire; PCS: pain catastrophizing scale.

Pearson correlation coefficients among the DAS28-CRP, PDQ, PSEQ, and PCS are shown in Figure 3. PSEQ had a significant positive correlation only with PCS (*r* = −0.345, *P* < 0.05). There was no significant correlation between PSEQ and DAS28-CRP.

There were significant positive correlations between the EQ-5D-5L and all items of the PSEQ (*P* < 0.05) (Table 5). The score on question 7 of the PSEQ (“I can cope with my pain without medication.”) was significantly lower than that on all other questions (*P* < 0.05) (Table 5). For the score on question 7 of the PSEQ, patients who used oral analgesics (2.48 ± 1.73, range; 0–6) had significantly lower scores than those who did not (3.45 ± 1.67, range; 0–6) (*P* < 0.018).

## 4. Discussion

Multiple previous studies have reported clinical and imaging remission in patients with RA, but few studies have focused on functional remission [4, 30]. In the present study, we investigated important factors associated with QOL in patients with RA, and our findings suggest that the disease duration, PDQ, PSEQ, PCS, use of oral steroids, and use of oral analgesics play important roles in achieving functional remission. Among these factors, the use of oral analgesics and PSEQ scores had particularly strong associations with QOL in patients with RA.

There are numerous tools to measure RA activity available for use [31]. The DAS28-CRP is widely used to evaluate RA activity and is a useful tool for assessing patients with RA [32]. The DAS28-CRP has several advantages: it is simple and quick, is useful for evaluating disease activity, and correlates with radiological progression [33, 34]. However, the disadvantages of the DAS28-CRP are its exclusion of the ankle and foot joints and less stringent remission criteria compared with the Simplified Disease Activity Index and Clinical Disease Activity Index [35, 36]. In addition, up to 40% of patients with RA demonstrated a progressive erosive disease detected by magnetic resonance imaging (MRI) despite DAS28-CRP improvement or EULAR remission [37]. Thus, other assessments should be included in the evaluation of patients with RA, such as the PSEQ and EQ-5D-5L, to provide a more complete description of the patient's overall recovery.

Pain in patients with RA is traditionally thought to be nociceptive pain of inflammatory origin [38]. However, 12.5% of patients with RA who achieve DAS28-CRP remission have clinically significant pain, and this pain may be neuropathic [6, 39]. Moreover, patients with RA who have medium and high PDQ scores have worse indicators of anxiety, depression, disability, HRQOL, pain, and fatigue [40]. Therefore, only utilizing the DAS28-CRP for the assessment and treatment of RA disease activity and only treating nociceptive pain may fail to improve QOL. Indeed, the assessment and treatment of pain (both nociceptive and neuropathic) with the goal of clinical remission are necessary for improving QOL.

In this study, psychosocial factors such as pain catastrophizing (PC) and pain self-efficacy (PSE), represented by the PCS and PSEQ scores, respectively, influenced the patients' QOL. Bandura [41] defined self-efficacy as “people's judgments of their capabilities to organize and execute courses of action required to attain designated types of performances.” PSE is patients' confidence that they can achieve their goals despite their pain [42]. PSE is associated with pain severity, pain intensity, negative psychological factors, and disability [9, 43–45]. In addition, high PSE is associated with a better QOL [9], and PSE is an important factor to consider for patients with RA [46].

PC is defined as “a set of exaggerated and negative cognitive and emotional schema brought to bear during actual or anticipated painful stimulation” [47]. PC is associated with pain intensity, depression, and anxiety [48]. One study found that one-fourth of patients with RA have high PCS, despite biotherapy [49]. Thus, PSE and PC are associated with pain and QOL, and pain management that can both increase PSE and reduce PC may ultimately improve QOL. One such strategy is acceptance and commitment therapy (ACT), which is a form of cognitive behavioural therapy [50]. ACT reduces pain intensity and increases self-efficacy [51, 52]. ACT also reduces PC and depression [50, 53]. Therefore, ACT would be useful for improving QOL in patients with RA.

In the present study, the EQ-5D-5L score was correlated with DAS28-CRP, PDQ, PSEQ, and PCS scores. PSEQ was not correlated with DAS28-CRP. Moreover, the multiple regression analysis revealed that PSEQ was an independent predictor of QOL for patients with RA. This provides evidence that assessing only DAS28-CRP and treating RA disease activity without considering PSEQ are insufficient for improving QOL. PCS was also correlated with DAS28-CRP, PDQ, and PSEQ. The correlation between PCS and PDQ was stronger than the correlation between PCS and DAS28-CRP or PSEQ. Therefore, the assessment and treatment of pain (both nociceptive and neuropathic) could reduce PCS and lead to an improvement in QOL.

In a previous report, a higher daily dose of oral steroids was associated with a low QOL [54]. In this study, the use of oral steroids was significantly associated with low EQ-5D-5L scores. Past reports have described the effectiveness of methotrexate with step-down oral steroids for the initial treatment of RA [55]. It may be better for a patient's QOL to avoid long-term administration of oral steroids if clinical remission could be achieved and maintained without use of oral steroids.

The use of oral analgesics was significantly associated with low EQ-5D-5L scores in this study. A previous study indicated that therapy with sDMARDs in combination with NSAIDs was associated with depression in patients with RA [56]. Depression is associated with PC and reduced QOL [48]. In this study, 19 of 25 patients who used oral analgesics also used NSAIDs, and 18 of those 19 patients were being treated with sDMARDs. Currently, therapy utilizing sDMARDs and bDMARDs represents the primary treatment for RA [57]. However, pain control with only NSAIDs may fail to achieve sufficient QOL in patients with RA. Therefore, other pain control measures without NSAIDs may be needed.

Concerning RA-related operations, there was no significant difference in QOL between patients who had and had not undergone one or more RA-related operations. Although the history of an RA-related operation appears to have no association with QOL, this also indicates that patients who have undergone an RA-related operation can achieve a QOL equivalent to those who did not require an operation.

The scores on question 7 of the PSEQ were significantly lower than the scores on the other items. Moreover, the scores on Question 7 of the PSEQ were significantly lower for patients who were taking oral analgesics than those for patients who were not. This could indicate a possible mechanism, such that the use of oral analgesics was associated with the patient's low feelings of self-efficacy, resulting in a lower QOL. Therefore, pain control with oral analgesics may provide less improvement in QOL, and rheumatologists should carefully consider the possibility of surgical intervention.

In this study, long disease duration and older age were risk factors for poor QOL, which is consistent with previous studies [58, 59]. Long disease duration is an important factor that increases symptoms of depression in patients with RA [60], and depression negatively impacts QOL [61]. In addition, elderly individuals usually have a poorer QOL [62], and they are more likely to feel pain than younger patients [63]. Pain and QOL are closely related, as described above. However, disease duration and age are factors that cannot be controlled. Therefore, elderly patients with RA and those with long disease duration may require a distinct overall treatment model.

The main strength of this study was the number of new variables studied in relation to QOL, which was assessed using the EQ-5D-5L. This study also has some limitations. First, the study population was relatively small, with a low number of male patients. It was a single-center study; therefore, a multicenter study may be needed to examine a large, diverse group of patients in the future. Second, almost all of the patients in this study had a well-controlled DAS28-CRP (mean score: 1.3 ± 0.9; range: 1.0–5.3). Therefore, it may not be a fully representative patient group. However, the majority of patients with RA achieve well-controlled RA disease activity due to the dramatic changes in RA treatment over the past 20 years [64]. Third, the patients' history of major depression was not investigated, and depression is expected to reduce QOL. Finally, this study obtained only cross-sectional data. Therefore, a prospective study may be needed in the future.

## 5. Conclusions

PSEQ scores and the use of oral analgesics are independent predictors of QOL in patients with RA. Pain control with only oral analgesics may lead to reduced QOL. Using only the DAS28-CRP for the assessment and treatment of RA disease activity may not adequately support the patient's QOL during RA treatment. Therefore, various pain management strategies, including surgical treatment, may be considered for the treatment of RA. Furthermore, the PSEQ may be included in the assessment of the efficacy of RA treatments.

## Figures and Tables

**Table 1:** Participant characteristics.

Number of patients	85

Sex (males, females; *n* (%))	11 (14.9), 74 (85.1)
Age (years; mean ± SD, range)	63.0 ± 12.3, 17–85
BMI (kg/m^2^; mean ± SD, range)	22.3 ± 4.0, 14.7–38.5
Disease duration (months; mean ± SD, range)	140.1 ± 116.5, 7–582
History of any RA-related operation, *n* (%)	40 (46.0)
Number of RA-related operations (mean ± SD, range)	0.9 ± 1.3, 0–6
Use of MTX, *n* (%)	55 (63.2)
Use of bDMARDs, *n* (%)	46 (52.9)
Use of oral steroids, *n* (%)	9 (10.3)
Use of oral analgesics, *n* (%)	25 (29.4)
MMP-3 levels (ng/mL; mean ± SD, range)	63.4 ± 45.1, 12.8–311.9
EQ-5D-5L (mean ± SD, range)	0.8096 ± 0.1570, 0.1722–0.9384
DAS28-CRP (mean ± SD, range)	1.3 ± 0.9, 1.0–5.3
PDQ (mean ± SD, range)	5.3 ± 4.5, 0–18
PSEQ (mean ± SD, range)	42.8 ± 13.0, 9–60
PCS (mean ± SD, range)	15.5 ± 11.2, 0–40

SD: standard deviation, BMI: body mass index, RA: rheumatoid arthritis, MTX: methotrexate, bDMARDs: biological disease-modifying antirheumatic drugs, MMP-3: serum matrix metalloprotease-3, EQ-5D-5L: the European Quality of Life questionnaire, five dimensions, five levels, DAS28-CRP: disease activity score based on the 28-joint assessment–C-reactive protein, PDQ: painDETECT questionnaire, PSEQ: pain self-efficacy questionnaire, PCS: pain catastrophizing scale.

**Table 2:** Type of oral analgesics and use of sDMARDs in patients using oral analgesics.

	Number of patients who use oral analgesics	Number of patients who also use sDMARDs among patients using oral analgesics
*N* (%)	25 (29.4)^*∗*^	23 (92)^†^
Type of oral analgesics		

NSAIDs, *n* (%)		
Acetaminophen, *n* (%)	3 (12)	3 (100)
Pregabalin, *n* (%)	3 (12)	2 (67)

sDMARDs: synthetic disease-modifying antirheumatic drugs, NSAIDs: non-steroidal anti-inflammatory drug. ^*∗*^Percentages in this column represent the percentage of the whole patient sample (*N* = 85); †percentages in this column represent the percentage of the patients who take oral analgesics or each specific type of oral analgesic.

**Table 3:** Independent *t*-tests between EQ-5D-5L and studied variables.

Parameters	Number of patients *n* (%)	EQ-5D-5L				*P* value
		Mean	Median	Standard deviation	Range	
*Sex*						0.183
Female	74 (87.1)	0.8008	0.8406	0.1595	0.1722–0.9384	
Male	11 (12.9)	0.8686	0.9384	0.1303	0.5960–0.9384	

*RA-related operations*						0.055
Absence	45 (52.9)	0.8403	0.8973	0.1205	0.4168–0.9384	
Presence	40 (47.1)	0.7750	0.8365	0.1854	0.1722–0.9384	

*Use of MTX*						0.175
Absence	30 (35.3)	0.7782	0.8324	0.1666	0.2466–0.9384	
Presence	55 (64.7)	0.8267	0.8973	0.1503	0.1722–0.9384	

*Use of bDMARDs*						0.261
Absence	46 (54.1)	0.8305	0.8406	0.1267	0.4168–0.9384	
Presence	39 (45.9)	0.7919	0.8555	0.1782	0.1722–0.9384	
*Use of oral steroids*						0.032
Absence	76 (89.4)	0.8299	0.8893	0.1358	0.1722–0.9384	
Presence	9 (10.6)	0.6383	0.7026	0.2213	0.2466–0.9384	

*Use of oral analgesics*						0.001
Absence	60 (70.6)	0.8456	0.8973	0.1235	0.4168–0.9384	
Presence	25 (29.4)	0.7233	0.7752	0.1944	0.1722–0.9384	

EQ-5D-5L: the European Quality of Life questionnaire, five dimensions, five levels, RA: rheumatoid arthritis, MTX: methotrexate, bDMARDs: biological disease-modifying antirheumatic drugs.

**Table 4:** Summary of the multiple regression analysis between the EQ-5D-5L and variables of interest.

	B	SE B	Β	*P* value
Age	−0.001	0.001	−0.074	0.414
Disease duration	0	0	−0.159	0.122
Use of oral steroids	−0.068	0.055	0.134	0.220
Use of oral analgesics	−0.085	0.031	−0.248^*∗*^	0.008
DAS28-CRP	−0.006	0.017	−0.033	0.730
PDQ	−0.005	0.003	−0.139	0.162
PSEQ	0.003	0.001	0.233^*∗*^	0.019
PCS	−0.003	0.001	−0.207	0.056
*R* ^2^ = 0.371				

EQ-5D-5L: the European Quality of Life questionnaire, five dimensions, five levels; MTX: methotrexate; DAS28-CRP: the disease activity score based on the 28-joint assessment–C-reactive protein; PDQ: painDETECT questionnaire; PSEQ: pain self-efficacy questionnaire; PCS: pain catastrophizing scale. ^*∗*^Significant difference between the EQ-5D-5L and studied variable (*P* < 0.05).

**Table 5:** Comparisons between individual items of the PSEQ.

Parameters	Score				*P* value between Q7 and each item (Tukey's test)	Correlation between EQ-5D-5L and each item (Pearson correlation)
	Mean	Median	Standard deviation	Range		
Q1	4.3529	5.0	1.5408	0–6	*P* < 0.001	*r* = 0.348, *P*=0.001
Q2	4.4471	5.0	1.5237	0–6	*P* < 0.001	*r* = 0.383, *P* < 0.001
Q3	4.7529	5.0	1.3085	0–6	*P* < 0.001	*r* = 0.228, *P*=0.036
Q4	4.4235	5.0	1.4005	0–6	*P* < 0.001	*r* = 0.271, *P*=0.012
Q5	4.2941	5.0	1.6462	0–6	*P* < 0.001	*r* = 0.437, *P* < 0.001
Q6	4.5529	5.0	1.4268	1–6	*P* < 0.001	*r* = 0.379, *P* < 0.001
Q7	3.1647	3.0	1.7379	0–6	*P*=1	*r* = 0.337, *P*=0.002
Q8	4.0706	4.0	1.6315	0–6	*P*=0.044	*r* = 0.366, *P*=0.001
Q9	4.4418	5.0	1.4662	0–6	*P* < 0.001	*r* = 0.404, *P* < 0.001
Q10	4.3176	5.0	1.4817	0–6	*P*=0.001	*r* = 0.359, *P*=0.001

PSEQ: pain self-efficacy questionnaire; Q: question. EQ-5D-5L: the European Quality of Life questionnaire, five dimensions, five levels.

## Data Availability

The data used to support the findings of this study are available from the corresponding author upon request.
